# A Price Paid for Our Internal Strife: Escalated Intragroup Aggression and the Evolution of Ingroup Derogation

**DOI:** 10.3389/fpsyg.2016.01453

**Published:** 2016-09-22

**Authors:** Qi Wu, Wang Liu, Chen Li, Xiongfeng Li, Ping Zhou

**Affiliations:** Cognition and Human Behavior Key Laboratory of Hunan Province, Department of Psychology, Hunan Normal UniversityChangsha, China

**Keywords:** ingroup derogation, intragroup aggression, threat management system, evolution, Penna model

## Abstract

From evolutionary reasoning, we derived a novel hypothesis that ingroup derogation is an adaptation to a special ecological condition in which the greater threat of aggression is incurred by ingroup members. This hypothesis was tested and supported across five studies. Specifically, the computational modeling found that ingroup derogation could easily evolve if the chance of death incurred by intragroup conflicts was no less than 10%. Further behavioral experiments on Chinese participants showed that the ingroup derogation mechanism responded to heuristic social category cues and it responded more strongly when participants subjectively felt more vulnerable to interpersonal aggression, or when there were contextual cues of aggression in the immediate environment. Additional results showed that Chinese participants responded more strongly to aggression cues originating from ingroup members and that they endorsed more ingroup derogation attitudes even when the ingroup and outgroup members were both displaying cues of aggression. In addition, the results also revealed that the Chinese participants perceived more intentions of aggression from ingroup members than from outgroup members even in the absence of any clear signs of those intentions, and such a bias was positively correlated with ingroup derogation attitudes. Taken together, these results suggest that ingroup derogation is related to the evolved response of intragroup aggression management system.

## Introduction

Ingroup derogation, also called outgroup favoritism, is a preference for outgroup members relative to one’s ingroup members (Jost et al., 2002; Ma-Kellams et al., 2011; Zhao et al., 2012; March and Graham, 2015; Wu et al., 2015). Both history and mainstream psychology demonstrated a tendency of favoring members of one’s own social group over those belonging to a different social group (i.e., ingroup favoritism, or ingroup bias; e.g., Bernhard et al., 2006; Navarrete and Fessler, 2006; Ruﬄe and Sosis, 2006; Rand et al., 2009; Yamagishi and Mifune, 2009). However, studies also documented the counterintuitive phenomenon of ingroup derogation among diverse groups. Specifically, individuals from minority or inferior groups may harbor ingroup derogation attitudes (Jost et al., 2002; Livingston, 2002; Rudman et al., 2002; Uhlmann et al., 2002; Ashburn-Nardo et al., 2003; March and Graham, 2015). Studies on the black sheep effect also showed that individuals derogated deviant ingroup members more negatively compared with their outgroup counterparts (Marques et al., 1988; Lewis and Sherman, 2010; Pinto et al., 2010; Reese et al., 2013). In addition, ingroup derogation was reported to be particularly prevalent in East Asian cultures (Hewstone and Ward, 1985; Heine and Lehman, 1997; Endo et al., 2000; Snibbe et al., 2003; Cuddy et al., 2009; Ma-Kellams et al., 2011). For example, researchers found that the Chinese were more inclined to cooperate with outgroup members (Wu et al., 2015), and they perceived the faces and names of outgroup members as more beautiful and better (Zhao et al., 2012). It was also reported that the Chinese implicitly associated European Americans with more positive traits than their own ethnic group (Ma-Kellams et al., 2011).

Few studies have examined the proximate mechanism of ingroup derogation. Since the bulk of the literature has focused on the minority groups, the theories mainly tried to explain the ingroup derogation found among inferior social groups (Rubin and Hewstone, 2004; Jost et al., 2007; Umphress et al., 2008). Theories also have proposed that subjects derogated deviant ingroup members because the ingroup norms were undermined (Pinto et al., 2010).

Although these theories have received some empirical support, they possess several limitations as an integral theoretical framework to account for all forms of ingroup derogation. According to these theories, ingroup derogation should be limited to dimensions that are status relevant or to members that are deviants and should not exist among majority group members. However, ingroup derogation has mainly been observed in East Asian cultures, in which the participants were not the minorities or deviants but the majorities (e.g., Hewstone and Ward, 1985; Zhao et al., 2012; Wu et al., 2015), and they seemed to possess a general, status irrelevant, and pervasive negative posture toward ingroup members (e.g., Ma-Kellams et al., 2011; Zhao et al., 2012; Wu et al., 2015).

Ma-Kellams et al. (2011) tried to explain these discrepancies by the dialectic theory. They proposed that individuals with an East Asian culture background are inclined to appraise both the bad and good for the same object, but Westerners mainly see the good. This theory can explain why the criteria of appraisal for East Asians are stricter than Westerners, but it cannot explain why lower ratings were still assigned to ingroup members when the participants held the same dialectical belief toward ingroup and outgroup members (Zhao et al., 2012; Wu et al., 2015). In sum, currently, researchers cannot well explain ingroup derogation in terms of proximate cause.

### The Ultimate Mechanism of Ingroup Derogation

Not only the causal origins of ingroup derogation are still unclear, the existence of ingroup derogation is also a paradox in an evolutionary sense. Numerous studies have documented the necessity of group living: individuals who preferred ingroups should have been favored by natural selection, whereas individuals displaying ingroup disfavoring tendencies should be eliminated from the gene pool over time (for review, see Brewer, 2007; Van Vugt and Park, 2009; Schaller and Neuberg, 2012; Cottrell and Park, 2013). Therefore, from the evolutionary perspective, favoritism toward outgroups is not considered as adaptation but should be considered as maladaptation. This makes it difficult to explain the prevalence and persistence of ingroup derogation.

In previous study, we derived a novel hypothesis that the mechanism of ingroup derogation is related to the evolved response of behavioral immune system, and it is specialized to deal with an ecological condition in which a greater threat of diseases is incurred by ingroup members (Wu et al., 2015). This hypothesis can partially explain why we dislike ingroup deviants and why East Asians derogate their ingroups. Preliminary evidence did support this hypothesis (Wu et al., 2015). However, this hypothesis cannot account for the ingroup derogation found in minority groups, nor does it take the threat of interpersonal aggression into consideration.

Interpersonal aggression is a recurrent threat faced by our ancestors (Van Vugt and Park, 2009; Schaller and Neuberg, 2012; Cottrell and Park, 2013). Since intergroup interactions are much more hostile and violent than within-group interactions, the threat of interpersonal aggression is mainly assumed to be manifested in the form of intergroup aggression (Schaller and Neuberg, 2012). Theories also have proposed that it is the threat of intergroup aggression that has shaped the mechanism of ingroup favoritism (Neuberg et al., 2011; Schaller and Neuberg, 2012).

However, regardless of how peaceful the intragroup interactions are, resources that are essential to one’s reproductive fitness (such as food, social status, and available mates) are still limited. Therefore, conflicts within the groups are inevitable. Since aggression can help an individual to gain social status or resources, or to protect oneself and one’s kins (Buss and Duntley, 2006), when the internal conflicts escalate, intragroup aggression will certainly become an acceptable option (LeBlanc, 2014). If somehow the resources are getting depleted (e.g., the occurrence of a famine, or the population density increases to approach the carrying capacity of the habitat), the intragroup conflicts would be greatly exacerbated (Stauffer, 2007; Ellis et al., 2009). Under such circumstances, aggression toward one another may become the usual condition. Considering the majority of social interactions occur within the boundary of ingroups (Schaller and Neuberg, 2012), then at that time, statically, the ingroup members may become more dangerous and hostile than outgroups, and it even may make them to be the major threat (i.e., the ingroup members would have higher mathematical expectations to cause interpersonal harm than the outgroup members). When that happens, it would be more adaptive to derogate, to fear, to hate, and to avoid the ingroup members than to bond with them, and a favoritism toward outgroup members would help our ancestors to abandon their original groups and to associate with other groups in order to find a better social group or more favorable habitats. If such situations occurred recurrently in the evolutionary history of the human race, then our ancestors probably had evolved psychological mechanisms to facilitate the response of favoring outgroup members over ingroup members under particular ecological conditions (i.e., under conditions in which the greater threat of aggression was incurred by ingroups instead of outgoups).

Historical records and results from anthropological studies suggest that such an assumption is plausible. Living in the East African Valley of Pleistocene, our ancestors were constantly facing the problems of acquiring enough resources to survive and reproduce (Buss, 1999; Chang et al., 2011). Famine, warfare, and diseases were the main challenges that our ancestors lived with and they are still the problems haunting our societies even today (Buss, 1999; Quinlan, 2007; Van Vugt and Park, 2009; Schaller and Neuberg, 2012; Cottrell and Park, 2013). Such environmental varieties make it quite possible for our ancestors to face the ecological condition in which ingroup investment is not optimal and should be reduced (Thornhill and Fincher, 2014). Simulation studies also suggest that ingroup derogation would be evolved if ingroup cooperation has collapsed or it is more beneficial to cooperate with outgroup members (Fu et al., 2012; Brown et al., 2016).

The proposed hypothesis may partially explain why now ingroup derogation is found to be prevalent among East Asians. It suggests that these participants are just responding to the heuristic cues which indicate that the intragroup competition and aggression has been greatly escalated. For example, the Chinese society has a long history of “internal strife” and it has long been characterized by a culture of “keen to fight their own people” (Boyang, 1992). The recent history of China also has been characterized by long time of civil war and internal turmoil (Li, 2012). Not to mention the unprecedented historical event of Cultural Revolution (i.e., a sociopolitical movement that took place in China from 1966 to 1976). This political turbulence sent the whole China into great turmoil, brought political persecution on a whole nation scale, and almost “turned the son against his father, turned the husband against his wife” (Yan and Gao, 1996). In addition, for mainland Chinese, their population density is significantly higher than that of Western countries, but usually their per capita income is significantly lower^1^ and this is a valid ecological cue suggesting the population density is approaching the carrying capacity of the habitat (Stauffer, 2007; Ellis et al., 2009). Therefore, it is possible that these historical and environmental cues have jointly led the Chinese to perceive their ingroups as dangerous and aggressive through direct experiences or social learning, which subsequently triggers their ingroup derogation mechanism. Consistent with this argument, some survey data show that approximately 73% of Chinese are feeling that it is unsafe to live in their local area, approximately 78% of Chinese think the crime problem in their local areas is quite serious, and approximately 83% of Chinese worry about walking alone at night (Wang et al., 2002). In contrast, such numbers seems to be much lower in Western cultures. For example, in the U.S., only 11–13% people believe that the crime problem is quite serious in their local areas and only 40% of people worry about walking alone at night^2^.

The hypothesis may also account for the ingroup derogation found in minority groups. For example, an inferior social status of a group may suggest that the resources which are accessible to that specific group might be very limited, which can also exacerbate the intragroup conflicts. Thus, members of minority groups may also endorse the ingroup derogation attitudes to escape from the dangerous ingroup members.

### The Current Study

Our argument seems plausible. However, such a hypothesis may only be “theoretically” plausible and its required conditions may never be satisfied. So whether it is possible to evolve an ingroup derogation tendency under our hypothesized conditions is still unknown. Computational modeling is a useful tool when we are trying to make accurate inferences about the behaviors of a complex system (Levinson and Gray, 2012). The sexual Penna model is a very successful model and it has been widely accepted in the field of biological evolution modeling due to its simplicity and predictability. This model is able to reproduce many features observed in real populations (e.g., the Eve effect; all alive individuals are descendants from one common ancestor), and it is the only Monte Carlo simulation technique that can fits the Gompertz law and the Azbel theory based on real demographic data (de Oliveira, 1998). Researchers have employed this model in numerous theoretical and empirical studies in biology and ecology, including the study of aging (e.g., Biecek and Cebrat, 2006; Periwal, 2013), the phenomenon of sympatric speciation (e.g., Sousa, 2004), the influence of medical care (Niewczas et al., 2000), the spreading of epidemics (He et al., 2005), the evolution of intelligence (e.g., Pan et al., 2005; He et al., 2007), social networks (e.g., Li and Maini, 2005), and language (Schwammle, 2007), and in the studies of the evolution of population dynamics of organisms (e.g., insects, fishes, wolves, and humans) in the laboratory or in the field (e.g., Makowiec, 1996; Penna and Stauffer, 1996; Giarola et al., 2006; de Oliveira et al., 2008, 2013; de Souza et al., 2012; dos Santos et al., 2012; Periwal, 2013), and so on (for a small review, see Stauffer, 2007). Due its success and validity in evolution modeling, we utilized the sexual Penna model to simulate the evolution of ingroup derogation in order to test our hypothesis in Study 1.

Results obtained through computational modeling are only the elaborate deductions of the theoretical model. An evolutionary hypothesis as we proposed needs to be tested both numerically and experimentally. After Study 1, five additional behavioral experiments (Studies 2, 3A and 3B, 4, 5) were also carried out. Specifically, in Studies 2 and 3, we tested whether the activation of ingroup derogation is related to the interpersonal aggression and whether this mechanism follows the smoke detector principle. In Study 4, we tested whether the Chinese participants perceive their ingroup members as more aggressive (more facial expressions of anger). Since the ingroup derogation is assumed to be a special adaptation designed to deal with a particular ecological condition in which ingroup members pose more threat of aggression than outgroup members, the perceived relative aggressiveness (between ingroups and outgroups) and ingroup derogation tendencies should be interconnected, and the mechanism of ingroup derogation also should respond more strongly to the cues of aggression mediated by ingroup members. These possibilities were tested in Study 5. In the present research, we mainly focused on the ingroup derogation among mainland Chinese.

## Study 1

In Study 1, we tested our hypothesis by utilizing a computational biological model. Specifically, we explored the theoretical boundaries of our hypothesis and examined whether it is possible to evolve the ingroup derogation tendency when the intragroup aggression becomes a greater threat than the threat of intergroup aggression. Previous studies on ingroup favoritism have shown that participants incline to affiliate with ingroups but to avoid outgroups (Van Vugt and Park, 2009; Schaller and Neuberg, 2012; Cottrell and Park, 2013). Therefore, in Study 1, we used the avoidance tendency as the measure of individuals’ preference for a specific group.

### The Model

The sexual Penna model (Sá Martins and Stauffer, 2001) was employed. The model was slightly modified in order to simulate virtual lives on a virtual continent (for a similar procedure, see He et al., 2005, 2007; Sousa, 2004; Pan et al., 2005). Here we only describe the necessary background and our modifications due to limited spaces. See the Supplementary Material for the extra detail of this model.

#### Virtual Individuals and Their Habitat

The sexual Penna model describes the aging and reproduction process of virtual individuals. In this model, each year, virtual individuals can grow, get sick, and reproduce (i.e., just like the real organism). The phenotype of each individual is controlled by its chromosomes. These chromosomes are represented by one pair of bit-strings (size A_max_) which are inherited from its parents. Virtual individuals die when they have acquired T diseases, otherwise they can live for A_max_ years at most. In our model, virtual individuals were represented as the same way as the sexual Penna model, and they also could grow, get sick, reproduce, and die as in the sexual Penna model. These virtual individuals lived on a virtual continent, a square lattice (L × L sites) with periodic boundary conditions. Virtual individuals were living on the sites of this continent and each site could only be taken by one individual. Resource with a maximum of Re_max_ could be carried by each site. Each year, the individual consumed the resource at rate of Re_con_. It died if it could not gain access to that resource. Each year, for the empty sites, resources increased at rate of Re_inc_.

Individuals on this continent were living in social groups. To encode their attitudes toward ingroup and outgroup members, two extra chromosomes (two pairs of bit-strings, size A_max_) were added in our model. These two chromosomes determined the avoidance tendency toward ingroup or outgroup members (i.e., F_in_: ingroup members; F_out_: outgroup members). If F_in_ > F_out_, it means individual prefers outgroups, otherwise it values ingroups over outgroups or treat them equally.

Each year, a mature female (age ≥ R) randomly chose a mature male among her eight neighboring sites to reproduce an offspring. Females who failed to find such mates could not reproduce. Offspring’s chromosomes were constructed by randomly crossing the chromosomes of parents as described in the sexual Penna model. M mutations were introduced during this process. The offspring randomly joined one of the social groups of its parents.

#### Intragroup and Intergroup Aggression

Because resources on the virtual continent were limited, individuals who could not gain access to the necessary resource Re_con_ would try to take another site (by randomly choosing) among one of the suitable sites (among its eight neighboring sites, and resource ≥Re_con_) in order to survive. If the neighboring site was not occupied by another individual, the invader would simply take this empty site. But, if the neighboring site was occupied, conflicts might occur. The neighbors would defend themselves and fight back. The invader could avoid such conflicts according to its intragroup and intergroup attitudes. Invader avoided ingroup defenders with probability of F_in_ and avoided outgroup defenders with probability of F_out_. If invader successfully avoided a conflict, it could try to exploit other suitable sites again, otherwise it had to engage in a fight with the defender. The Invaders would have a probability of P_in_ or P_out_ to lose the battle (thus 1 - P_in_ or 1 - P_out_ to win the battle) according to defenders’ social group (P_in_ for ingroup members, P_out_ for outgroup members). Higher value in P_in_ indicates greater level of intragroup aggression, whereas higher value in P_out_ indicates greater level of intergroup aggression. Losers of such conflicts died.

#### Simulation Protocol

Simulations were initialized by randomly distributing *N*_0_ virtual individuals on a square lattice. Both the sex and the social group of a virtual individual were randomly initialized. The Health Bit-String was initialized with all the positions equal to zero. The contents of Ingroup and Outgroup Bit-String were also randomly generated. To make the model tractable, following parameters were fixed at the reasonable values: L = 100,N_0_ = 7500,A_max_ = 100,Re_max_ = 40,Re_con_ = 2,Re_inc_ = 8,R = 11,T = 7,M = 1. Since for our ancestors the mean number of group members in a group was about 150 (Dunbar, 1993), the number of social groups N in the population was initialized to be 50 (N_0_/N = 7500/50 = 150).

Simulations were carried out 20 times for a given set of parameters using different initial seeds for the random number generator, and were stopped when the virtual system had converged (i.e., the order parameters had stabilized around certain stationary values in each round of simulations, including the population size and the mean avoidance tendencies toward ingroup or outgroup members). In each simulation, the mean of the whole virtual population in each time step was recorded as the data. Then the mean of the data from last 10000 time steps were calculated for each simulation and the average values of these calculated means of 20 simulations were taken as the results for a given set of parameters.

### Results and Discussion

**FIGURE 1:**
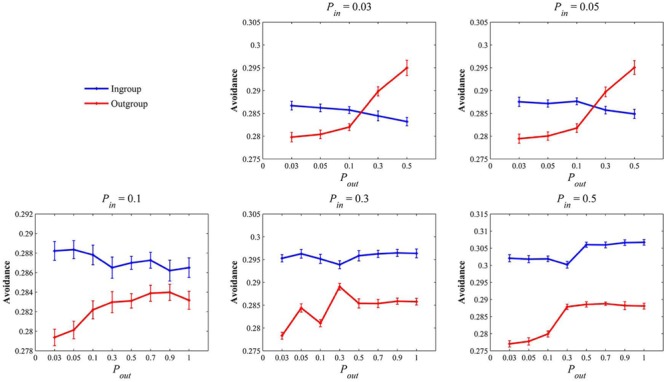
**The mean ingroup and outgroup avoidance tendency of the population.** Error bars represent the three standard errors.

With all parameter values, the virtual population survived on the lattice, with a very stable population size around 7300 (see the Supplementary Material for more detailed results). When level of intragroup aggression was set to low (P_in_ = 0.03 or 0.08), the results were very similar (see **Figure 1**). Virtual population preferred outgroups when intergroup interactions were relatively peaceful (P_out_ = 0.03,0.05, and 0.1). But this relationship was reversed when intergroup interactions became quite violent (P_out_ = 0.3 and 0.5). This is consistent with the prediction made by theories of ingroup favoritism (i.e., ingroup favoritism would be evolved when outgroups were more dangerous; see Schaller and Neuberg, 2012).

We raised the intragroup aggression factor to a higher level (P_in_ = 0.1). Under this circumstance, the level of intragroup aggression was still low, but a rate of 10% deaths was enough to have significant impacts on fitness values (LeBlanc, 2014). The results showed that, under this circumstance, regardless of the level of intergroup aggression (P_out_ = 0.03 ∼ 1), virtual population would eventually evolve a mild ingroup derogation tendency (see **Figure 1**). Similar results were obtained when we raised intragroup aggression to more intense levels (P_in_ = 0.3 and 0.5). Results showed that ingroup-disfavoring tendencies were exaggerated as intragroup interactions were becoming more dangerous (see **Figure 1**).

In summary, Study 1 showed that, when intergroup interactions were relatively peaceful (P_out_ ≤ 0.1), a tendency to favor outgroups over ingroups would evolve. The results also showed that, if intragroup aggression escalated to a considerable level (P_in_ ≥ 0.1), evolution of ingroup derogation would be inevitable. Considering that the rate of 10% still has significant impacts on one’s reproductive fitness (LeBlanc, 2014), results of Study 1 suggest that, an intergroup bias of derogating the ingroups could be evolved under a broad range of conditions and such required conditions might be easily satisfied in the real world. Therefore, the results of Study 1 suggest that ingroup derogation might be an evolved psychological mechanism and it might be related to the escalated intragroup aggression.

## Study 2

Study 1 showed the possibility of evolution of ingroup derogation. However, results of the simulation study depend on their assumptions. Without some actual data, it is still hard to know whether ingroup derogation is an evolved mechanism. In Study 2, we tried to provide some experimental evidence to support our hypothesis in the first place.

According to our hypothesis, ingroup derogation is an evolved response of the intragroup aggression management system. Such an evolved threat management mechanism should follow the smoke detector principle and the functional flexibility principle (Van Vugt and Park, 2009; Neuberg et al., 2011; Schaller and Neuberg, 2012; Cottrell and Park, 2013).

The smoke detector principle dictates that a threat management system is deliberately calibrated to minimize false-negative errors, but with the inevitable consequence that it is prone to make false-positive errors. Such a system has to respond to heuristic cues which imply the presence of potential threats. According to this principle, ingroup derogation shouldn’t exclusively exist in actual social groups (e.g., Zhao et al., 2012). Mere social categorization alone—a heuristic cue that implies the differentiation between “us” and “them” (Tajfel et al., 1971; Bernstein et al., 2007)—should be sufficient to bring this bias. Minimal group paradigm categorizes people into artificially distinct groups on the basis of arbitrary criteria, such as whether they have a “red” personality type or a “green” personality type based on a bogus test (Bernstein et al., 2007), which provides group-categorization heuristics to one’s actual social group (Tajfel et al., 1971). In Study 2, by using the minimal group paradigm, we tested whether Chinese participants would show ingroup derogation when cues denoting one’s group (artificial labels) were only heuristically associated with one’s actual social group (i.e., smoke detector principle). Since previous study has shown that ingroup derogation attitudes among actual social groups can be shown in a form of facial beauty appraisal task (Zhao et al., 2012), we employed the same measure of ingroup derogation attitudes (to rate the degree of beauty for a specific face) in Study 2.

The functional flexibility principle indicates the psychological mechanisms of threat management are sensitive to individuals’ apparent vulnerability to specific threats and modulate threat-minimizing responses accordingly. As a specific form of threat management system, the mechanism of ingroup derogation should also respond more strongly when individuals become more vulnerable or just perceive themselves to be more vulnerable. Specifically, for the Chinese participants who have more chronic concerns about interpersonal aggression and harm, their perceived level of intragroup aggression should be stronger than the usual. Although their perceived level of intergroup aggression also should be increased, considering that participants were responding to a special ecological condition in which the greater threat was posed by ingroup members, their perceived differences between the level of intragroup and intergroup aggression actually should be enlarged. As a result, responses of ingroup derogation should become stronger for these participants. This prediction was tested in Study 2. Specifically, we also examined whether there was a positive association between the chronic concerns of interpersonal aggression and the degree of ingroup derogation in this study (i.e., functional flexibility principle).

### Method

#### Participants and Design

Sixty mainland Chinese undergraduate or postgraduate students (30 males and 30 females, mean age = 20.3 years, *SD* = 1.98) participated in this study for monetary compensation. This study was carried out in accordance with the recommendations of the IRB of the Institute of Psychology, Hunan Normal University, with written informed consent from all participants. All participants gave written informed consent in accordance with the Declaration of Helsinki.

A 2 (personality type: red, green) × 2 (category label: ingroup, outgroup) mixed-model experimental design was used, with personality type being the between-subjects factor and category label being the within-subjects factor.

#### Materials and Procedure

A bogus personality test that was identical to Wu et al. (2015) was used to create the minimal groups. The computer ostensibly analyzed participants’ responses after they completed the test. Participants were then informed that they had either a “red” or a “green” personality type.

The individual differences in chronic concerns of interpersonal aggression and harm was assessed by a questionnaire of belief in a dangerous world (BDW; Altemeyer, 1988; sample item: “There are many dangerous people in our society who will attack someone out of pure meanness, for no reason at all”). Higher score on this measure indicate greater extent of chronic concerns of interpersonal aggression and harm (in present study, α = 0.69).

Eighty gray-scale facial images of Chinese adults with neutral facial expressions were employed as the stimuli. These images had already been employed in previous studies (Zhao et al., 2012; Wu et al., 2015). These images, completely novel to all participants, consisted of two image sets matched on the degrees of beauty (Zhao et al., 2012) and acceptance (Wu et al., 2015). Each facial stimulus was presented in center of the screen. A label of personality type (red or green) was placed at the top of the background in order to label the face. The background color of the screen was set to be identical to the personality label (red or green). The two image sets were counterbalanced across background color (and its personality label) on a between-subjects basis.

Participants were instructed that they would take a computerized personality test at first. Participants were then instructed that they would view faces on the screen, and that the background color and the label displayed on the top of the screen would denote the target’s personality type. They were informed that their task was to rate the degree of beauty on a 10- point scale for these faces (1 = “not beautiful at all,” 10 = “extremely beautiful”). Each face was presented for 2000 ms. After participant’s response, a black screen appeared for a randomized duration from 2000 to 2500 ms. Sequence of the faces were randomized for each participant.

### Results and Discussion

**FIGURE 2:**
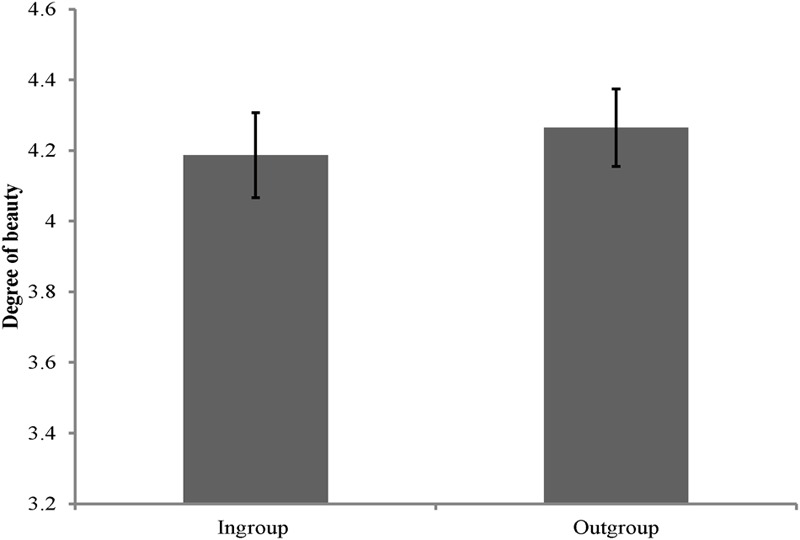
**Degree of beauty of faces labeled as ingroup members and outgroup members in Study 2.** Error bars represent standard errors.

The rating scores of the face appraisal task were subjected to a 2 (personality type: red, green) × 2 (category label: ingroup, outgroup) mixed-model analysis of variance (ANOVA). The main effect of category label was significant [*F*(1,58) = 6.03, *p* < 0.05, $\eta_{p}^{2}$ = 0.09], indicating that participants perceived the faces of outgroup members as slightly more beautiful (see **Figure 2**). However, the main effect of personality type [*F*(1,58) = 0.08, *p* > 0.05, $\eta_{p}^{2}$ = 0.001] and the interaction between category label and personality type [*F*(1,58) = 2.81, *p* > 0.05, $\eta_{p}^{2}$ = 0.05] were not significant. Therefore, participants showed ingroup derogation when the cues denoting one’s group were only heuristically associated with one’s actual social group membership.

Rating scores of outgroup members were then subtracted by the rating scores of ingroup members to create a composite score of ingroup derogation. A higher value on this score indicates a stronger tendency to derogate ingroup members (*M* = 0.08, *SD* = 0.25). Results showed that this composite score was positively correlated with BDW, *r* = 0.27, *p* < 0.05. These results indicated that there was positive association between the degree of ingroup derogation and the chronic concerns of interpersonal aggression and harm.

In sum, Study 2 directly replicated and extended the previous studies of Zhao et al. (2012) and Wu et al. (2015) by showing that Chinese participants perceived the faces of outgroups as more beautiful under minimal group situation and this ingroup-disfavoring tendency was also positively correlated with the chronic concerns of interpersonal aggression and harm. The results of Study 2 indicate the mechanism of ingroup derogation follows the smoke detector principle and functional flexibility principle. These results provide the first empirical evidence for our hypothesis that ingroup derogation is an evolved response of intragroup aggression management system.

## Study 3A

Study 2 found that the subjective feeling of vulnerability to interpersonal harm was positively associated with the attitude of ingroup derogation. However, according to the functional flexibility principle, an intragroup aggression management system—like the mechanism of ingroup derogation— should not only be activated on the basis of individual differences. When there are contextual cues of danger in the immediate environment, its activation also should become stronger in order to respond to the emergence of imminent threat.

For humans, the onset of darkness can arouse fear and anxiety (Grillon et al., 1997; Schaller et al., 2003a,b; Miller et al., 2010). It serves as a heuristic cue indicating vulnerability to physical danger since we rely heavily on vision to navigate physical and social landscapes and to avoid dangers lurking within those landscapes (Grillon et al., 1997). Being limited in visual input, such as in the ambient darkness condition, facilitates aggression against other individuals and may greatly increase our vulnerability to physical violence and other kinds of aggression (Page and Moss, 1976; Schaller and Neuberg, 2012; Jonason et al., 2013). Studies on intergroup cognition have also shown that ambient darkness is an ecological relevant contextual cue associated with vulnerability to aggression since it only activates aggression related stereotypes (Schaller et al., 2003a,b). In Study 3A, we examined the effect of ambient darkness on ingroup derogation attitudes. As mentioned above, ambient darkness is a dangerous situation in which we are more likely to be harmed by others. Thus, according to the functional flexibility principle, when the mainland Chinese are placed in the ambient darkness, the mechanism of the ingroup derogation should be more activated in order to deal with the enlarged vulnerability to physical harms. More importantly, since the Chinese are responding to a special ecological condition in which the greater threat of aggression is imposed by ingroup members, their elicited defense against intragroup aggression should be more pronounced than their elicited defense against intergroup aggression. Therefore, we expected to find an exaggerated ingroup derogation attitude for mainland Chinese under the ambient darkness circumstance.

### Method

#### Participants and Design

One hundred and twenty paid volunteers, all mainland Chinese undergraduate or postgraduate students (48 males and 73 females, mean age = 19.9 years, *SD* = 2.76), participated in this study. This study was carried out in accordance with the recommendations of the IRB of the Institute of Psychology, Hunan Normal University, with written informed consent from all participants. All participants gave written informed consent in accordance with the Declaration of Helsinki.

A 2 (personality type: red, green) × 2 (category label: ingroup, outgroup) × 2 (environmental setting: dark condition, light condition) mixed-model experimental design was used, with personality type and environmental setting being the between-subjects factors while category label being the within-subjects factor.

#### Materials and Procedure

The bogus personality test which was used to create minimal groups and the facial stimuli that were employed by this experiment was identical to those of Study 2.

Participants were seated in a windowless room that could be well-lit by electric lights. The participants in the light condition had to finish the study with overhead lights all turned on. Participants assigned to the dark condition had to finish this study with overhead lights all turned off. This left the room in total darkness except for the lights emitted by the computer screens.

The procedure of Study 3A was identical to that of Study 2, except that all participants were additionally instructed to rest for 2 min after they finished the bogus personality test. During this phase, participants in the dark condition were instructed to wear an eye mask to completely block the light. They were then instructed to remove the mask and finish the rest of experiment when the rest phase was over. Participants in light condition did not have to wear any eye masks. They were just instructed to rest for 2 min and then were instructed to finish the rest of experiment. Participants didn’t have to complete the BDW questionnaire.

### Results and Discussion

**FIGURE 3:**
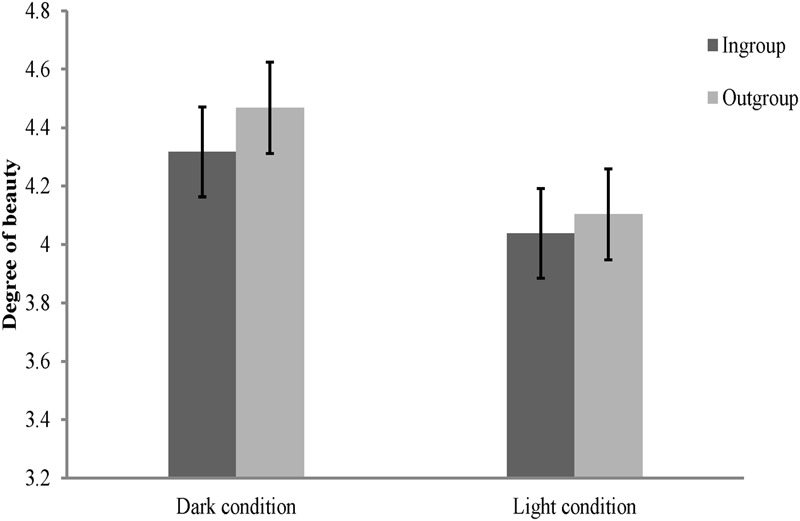
**Degree of beauty of faces labeled as ingroup members and outgroup members in Study 3A.** Error bars represent standard errors.

Rating scores for ingroup and outgroup members were subjected to a 2 (personality type: red, green) × 2 (category label: ingroup, outgroup) × 2 (environmental setting: dark condition, light condition) mixed-model ANOVA. Results showed that the main effect of category label [*F*(1,116) = 26.16, *p* < 0.01, $\eta_{p}^{2}$ = 0.18], and the interaction between category label and environmental setting [*F*(1,116) = 4.17, *p* < 0.05, $\eta_{p}^{2}$ = 0.04] were significant. The main effect of personality type [*F*(1,116) = 0.001, *p* > 0.05, $\eta_{p}^{2}$ < 0.001], the main effect of environmental setting [*F*(1,116) = 2.17, *p* > 0.05, $\eta_{p}^{2}$ = 0.02], the interaction between personality type and category label [*F*(1,116) = 0.24, *p* > 0.05, $\eta_{p}^{2}$ = 0.002], and the interactions of all these three independent variables [*F*(1,116) = 0.04, *p* > 0.05, $\eta_{p}^{2}$ < 0.001], were not significant. Consistent with Study 2, participants preferred outgroups over ingroups under all environmental settings [dark condition: *F*(1,116) = 25.62, *p* < 0.01, $\eta_{p}^{2}$ = 0.18; light condition: *F*(1,116) = 4.72, *p* < 0.05, $\eta_{p}^{2}$ = 0.04; see **Figure 3**]. However, effects of environmental setting were not significant for both ingroup and outgroup members [ingroup: *F*(1,116) = 1.64, *p* > 0.05, $\eta_{p}^{2}$ = 0.01; outgroup: *F*(1,116) = 2.72, *p* > 0.05, $\eta_{p}^{2}$ = 0.02].

To illustrate the interaction between category label and environmental setting, a composite score of ingroup derogation as described in Study 2 was created. We subjected this score to a 2 (personality type: red, green) × 2 (environmental setting: dark condition, light condition) independent ANOVA. The results showed that the main effect of personality type [*F*(1,116) = 0.52, *p* > 0.05, $\eta_{p}^{2}$ = 0.004], and the interaction between personality type and environmental setting [*F*(1,116) = 0.04, *p* > 0.05, $\eta_{p}^{2}$ < 0.001], were not significant. The main effect of environmental setting [*F*(1,116) = 4.18, *p* < 0.05, $\eta_{p}^{2}$ = 0.04] was significant, with participants showing more ingroup derogation in dark condition (*M* = 0.15, *SD* = 0.21) than in light condition (*M* = 0.07, *SD* = 0.25).

Collectively, results of Study 3A are consistent with our prediction that the vulnerability-connoting circumstance of ambient darkness would trigger an exaggerated attitude of ingroup derogation among mainland Chinese. Study 3A directly replicated and extended Study 2 by showing that the response of ingroup derogation mechanism could be exaggerated when individual were under realistic danger condition. These results indicate the mechanism of ingroup derogation follows the smoke detector principle and functional flexibility principle, which again supports our hypothesis that ingroup derogation is an evolved response of intragroup aggression management system.

Although previous studies have revealed the close linkage between ambient darkness and aggression (Page and Moss, 1976; Grillon et al., 1997; Schaller et al., 2003a,b; Miller et al., 2010; Jonason et al., 2013), the manipulation of ambient darkness as a contextual cue of aggression in Study 3A may still be subjected to some confounds. Thus, it is difficult for us to completely rule out other alternative explanations by Study 3A alone. To provide further confidence to the finding of Study 3A, we performed a follow-up study (Study 3B) in which we again examined the effects of contextual cue of aggression on ingroup derogation, but used a more rigorous method of threat priming.

## Study 3B

### Method

#### Participants and Design

One hundred and twenty paid volunteers, all mainland Chinese undergraduate students (60 males and 60 females, mean age = 20.35 years, *SD* = 1.56), participated in this study. This study was carried out in accordance with the recommendations of the IRB of the Institute of Psychology, Hunan Normal University, with written informed consent from all participants. All participants gave written informed consent in accordance with the Declaration of Helsinki.

A 2 (personality type: red, green) × 2 (category label: ingroup, outgroup) × 2 (threat priming: aggression salient, control) mixed-model experimental design was used, with personality type and priming being the between-subjects factors while category label being the within-subjects factor.

#### Materials and Procedure

The bogus personality test and the facial stimuli employed by Study 3B were identical to those of Study 2. However, since the effects of threat priming may not last very long and finishing the face appraisal task of Study 2 required a lot of time, the face appraisal task of Study 3B was replaced by a shorter version^3^ (Wu et al., 2015). In Study 3B, the task of the participants was to rate the degree of acceptance for these faces on an 8-point scale (“to what extent would you want to work together with the person shown on the screen in the next experiment”; 1 = “definitely not” to 8 = “definitely like to”). The stimulus presentation, rating, and counterbalance procedures were identical to Study 2 except that each face remained on the screen until a response was made and no blank screens were inserted between the trials.

Participants were instructed to finish the bogus personality test at first. Then the participants were required to complete another two unrelated tasks. In the first task, participants received the threat priming manipulation by watching short film clips with silenced sound tracks. Participants were asked to watch closely in order to answer questions about them. Participants in the aggression salient condition watched a 7-min film clip depicting severe interpersonal aggression (e.g., people were violently attacked by others), whereas participants in the control condition watched a 7-min film clip depicting several different accidents (e.g., car accidents, air crash). To ensure the validity of these two threat primes, a pilot study (*n* = 22) was carried out. Participants rated the danger levels of the threat of aggression, accidents, and diseases conveyed by these two threat primes and the dimensions of pleasantness and arousal of these two videos in 9-point scales. The results showed that the aggression salient prime mainly conveyed the threat of interpersonal aggression (aggression: *M* = 7.32, *SD* = 1.81; accident: *M* = 1.82, *SD* = 1.05; disease: *M* = 1.73, *SD* = 1.16), aggression vs. accident [*t*(20) = 11.85, *p* < 0.001], aggression vs. disease [*t*(20) = 12.56, *p* < 0.001], accident vs. disease [*t*(20) = 0.29, *p* > 0.05]. The control prime mainly conveyed the danger of accidents (aggression: *M* = 2.14, *SD* = 1.36; accident: *M* = 7.36, *SD* = 1.71; disease: *M* = 1.82, *SD* = 0.85), accident vs. aggression [*t*(20) = 9.94, *p* < 0.001], accident vs. disease [*t*(20) = 12.07, *p* < 0.001], aggression vs. disease [*t*(20) = 1.13, *p* > 0.05]. The results also showed that two primes were matched on the pleasantness (aggression salient: *M* = 2.05, *SD* = 1.43; control: *M* = 2.64, *SD* = 1.73; *t*(21) = -1.71, *p* > 0.05), arousal (aggression salient: *M* = 6.91, *SD* = 2.65; control: *M* = 6.77, *SD* = 2.52; *t*(21) = 1, *p* > 0.05), and the danger level of diseases [*F*(1,21) = 0.32, *p* > 0.05, $\eta_{p}^{2}$ = 0.02]. In addition, the aggression salient prime contained significant higher level of aggression threat than the control prime [*F*(1,21) = 109.52, *p* < 0.001, $\eta_{p}^{2}$ = 0.84], whereas the control prime depicted more dangerous accidents than the aggression salient prime [*F*(1,21) = 125.23, *p* < 0.001, $\eta_{p}^{2}$ = 0.86]. There was no significant difference between the danger level of the aggression threat depicted in the aggression salient prime and the danger level of the accidents depicted in the control prime [*t*(21) = -0.11, *p* > 0.05].

After the threat priming, each participants were asked to finish the face appraisal task. Then the participants were debriefed.

### Results and Discussion

**FIGURE 4:**
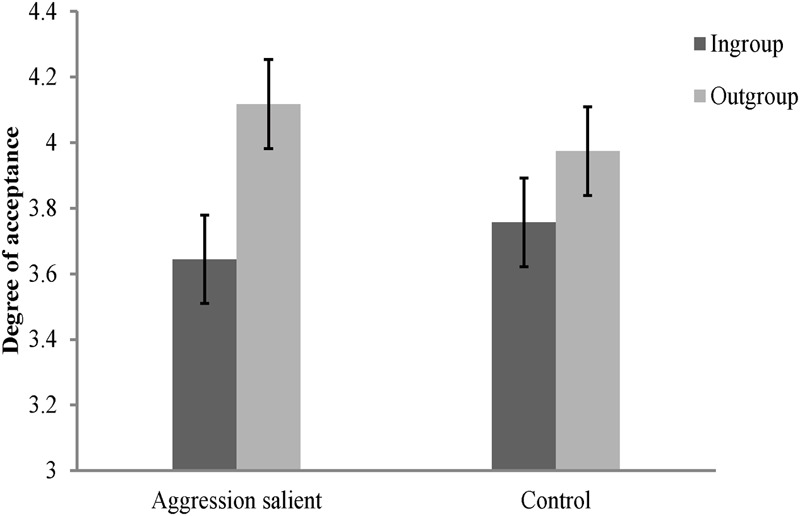
**Degree of acceptance of faces labeled as ingroup members and outgroup members in Study 3B.** Error bars represent standard errors.

The rating scores of the face appraisal task were subjected to a 2 (personality type: red, green) × 2 (category label: ingroup, outgroup) × 2 (threat priming: aggression salient, control) mixed-model ANOVA. The results showed that the main effect of category label [*F*(1,116) = 71.06, *p* < 0.001, $\eta_{p}^{2}$ = 0.38], and the interaction between category label and threat priming [*F*(1,116) = 9.88, *p* < 0.01, $\eta_{p}^{2}$ = 0.08] were significant. The main effect of personality type [*F*(1,116) = 0.36, *p* > 0.05, $\eta_{p}^{2}$ = 0.003], the main effect of threat priming [*F*(1,116) = 0.007, *p* > 0.05, $\eta_{p}^{2}$ < 0.001], the interaction between personality type and category label [*F*(1,116) = 0.51, *p* > 0.05, $\eta_{p}^{2}$ = 0.004], and the interactions of all these three independent variables [*F*(1,116) = 0.004, *p* > 0.05, $\eta_{p}^{2}$ < 0.001], were not significant. Consistent with Study 3A, further simple effect analysis revealed that the effects of threat priming were not significant for both ingroup and outgroup members [ingroup: *F*(1,116) = 0.35, *p* > 0.05, $\eta_{p}^{2}$ = 0.003; outgroup: *F*(1,116) = 0.57, *p* > 0.05, $\eta_{p}^{2}$ = 0.005]. But the results also showed that participants consistently derogated their ingroup members regardless of the type of threat priming they received [aggression salient: *F*(1,116) = 66.97, *p* < 0.001, $\eta_{p}^{2}$ = 0.37; control: *F*(1,116) = 13.97, *p* < 0.001, $\eta_{p}^{2}$ = 0.11; see **Figure 4**].

In order to clearly illustrate the source of the interaction between category label and threat priming, a composite score of ingroup derogation as described in Study 2 was created. We subjected this score to a 2 (personality type: red, green) × 2 (threat priming: aggression salient, control) independent ANOVA. The results showed that the main effect of personality type [*F*(1,116) = 0.52, *p* > 0.05, $\eta_{p}^{2}$ = 0.004], and the interaction between personality type and threat priming [*F*(1,116) = 0.01, *p* > 0.05, $\eta_{p}^{2}$ < 0.001], were not significant. The main effect of threat priming [*F*(1,116) = 9.74, *p* < 0.01, $\eta_{p}^{2}$ = 0.08] was significant, with participants showing greater ingroup derogation in aggression salient condition (*M* = 0.47, *SD* = 0.42) than in control condition (*M* = 0.22, *SD* = 0.46).

Again, the results of Study 3B indicated that when the situation signaled a heightened need to protect the self from aggression, participants endorsed stronger ingroup derogation attitudes. It directly replicated and extended previous studies (Wu et al., 2015) by showing that the threat of interpersonal aggression was also able to influence the ingroup derogation attitudes for Chinese participants. Since the threat priming materials were rigorously controlled and matched in many different dimensions, the results of Study 3B made it possible for us to reject the alternative explanations that cannot be rejected by Study 3A. Taken together, the results of Studies 3A and 3B consistently indicate that the mechanism of ingroup derogation follows the functional flexibility principle and there is a close linkage between the threat of interpersonal aggression and ingroup derogation.

## Study 4

The results of Studies 2 and 3 provided the direct evidence that the activation of ingroup derogation mechanism was closely related to the threat of aggression. However, these results only provided the indirect evidence to the part of our hypothesis that the greater threat of aggression was incurred by ingroup members. To directly test this part of our hypothesis, Study 4 was carried out.

In order to facilitate the adaptive responses to external threats, a threat management system — such as the proposed ingroup derogation mechanism — should be able to influence numerous downstream cognitive and emotional processes (Schaller and Neuberg, 2012). According to our hypothesis, the ingroup derogation mechanism was designed to deal with a special ecological condition in which the greater threat of aggression is brought by the ingroup members. If such hypothesis is true, this special ecological condition must have shaped our basic social perceptions as well. It will lead us to perceive our ingroup members as being more aggressive than the outgroup members in order to facilitate the ingroup derogation response.

Since directly asking the participants about their opinions of the aggressiveness of ingroup/outgroup members may be subject to social consent, a more indirect and implicit measure of aggressiveness was used by Study 4. To recognize potential aggression, individuals must recognize signs of that potential. The emotion of anger is assumed to prepare an organism to attack and harm (Fessler, 2010). Numerous studies have documented the close relationship between anger and aggression (e.g., Averill, 1983; Ramirez and Andreu, 2006). Studies also showed that the facial expression of anger directly conveys the intention of aggression and is a strong precursor to interpersonal aggression and violence (e.g., Ekman, 1982; Shariff and Tracy, 2011; Matsumoto et al., 2014). Therefore, the Chinese participants should be able to perceive greater anger in the ingroup faces than in the outgroup faces. Specifically, considering that a failure to identify an actual threat is generally more costly than the assumption of threat when none exists (i.e., smoke detector principle; Schaller and Neuberg, 2012), the mainland Chinese should have a general tendency to perceive greater anger in the faces of ingroup members than in the faces of outgroup members even in the absence of any clear signs of angry facial expressions. This prediction was tested in Study 4.

### Method

#### Participants and Design

Ninety-eight mainland Chinese undergraduate or postgraduate students (age 18–26; 67 males and 31 females) were paid to participate in this study. This study was carried out in accordance with the recommendations of the IRB of the Institute of Psychology, Hunan Normal University, with written informed consent from all participants. All participants gave written informed consent in accordance with the Declaration of Helsinki.

A 2 (personality type: red, green) × 2 (category label: ingroup, outgroup) mixed-model experimental design was used, with personality type being the between-subjects factors while category label being the within-subjects factor. Fifty participants were randomly assigned to the red personality type, while 48 participants were randomly assigned to the green personality type.

#### Materials and Procedure

The bogus personality test which was used to create minimal groups was identical to that of Study 2.

Forty facial stimuli from Study 2 were randomly chosen as the facial stimuli in this experiment. These faces were randomly divided into two sets, with 20 faces in each set. Twenty college students who did not participate in the formal studies rated the degree of beauty for these faces on a 10-point scale. A pairwise *t*-test showed that there was no difference for the degree of beauty between these two image sets (first set: *M* = 5.22, *SD* = 1.16; second set: *M* = 4.97, *SD* = 0.99), *t*(19) = 1.34, *p* > 0.05. These two image sets were then presented in this experiment in the same way as in Study 2.

Participants took the computerized personality test at first. They were then instructed that they would view faces on the computer screen and the background color and the label displayed on the top of the screen would denote the target’s personality type. They were told that, these facial images were taken when the targets were trying to conceal their real emotions by putting on a neutral face, thus there might be some clues on their faces to reveal their real emotional feelings (such as subtle expressions or micro-expressions). The task of participants was to rate the extent to which they believed the target was actually expressing (a) happiness, (b) sadness, (c) anger, and (d) fear on 9-point scales with endpoints 1 (not at all) and 9 (very much) (for a similar procedure, see Maner et al., 2005). The faces were presented one at a time and each face remained on the screen until the four emotion rating tasks were completed for that target. Sequence of faces were randomized for each participant. Sequence of these four emotion appraisal tasks were also randomized for each facial stimuli.

### Results and Discussion

**FIGURE 5:**
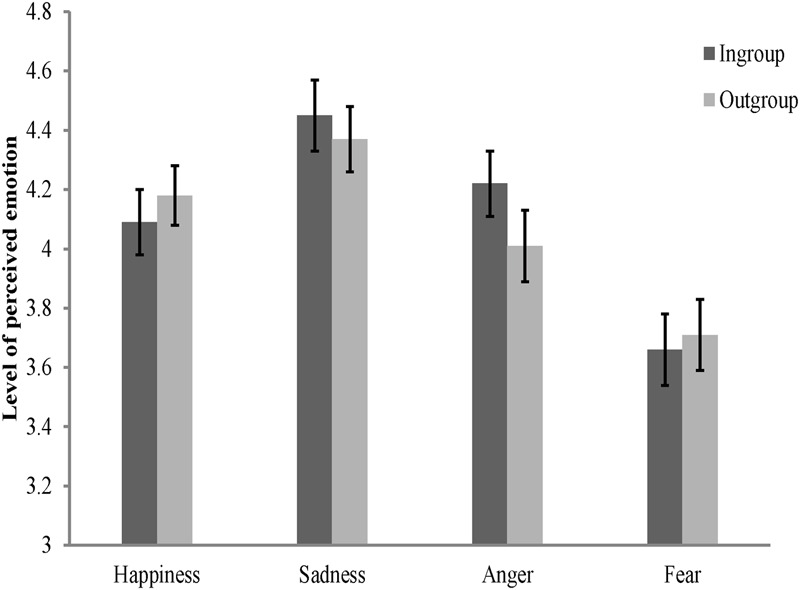
**Mean levels of perceived emotions in inroup and outgroup faces in Study 4.** Error bars represent standard errors.

Rating scores of four emotion appraisal tasks were separately subjected to four 2 (personality type: red, green) × 2 (category label: ingroup, outgroup) mixed-model ANOVAs. Results showed that, the main effect of category label [*F*(1,96) = 4.64, *p* < 0.05, $\eta_{p}^{2}$ = 0.05] was significant on the perception of anger, participants perceived greater anger in ingroup faces than in outgroup faces (see **Figure 5**). However, the main effect of personality type [*F*(1,96) = 1.67, *p* > 0.05, $\eta_{p}^{2}$ = 0.02] and the interaction between these two independent variables [*F*(1,96) = 0.07, *p* > 0.05, $\eta_{p}^{2}$ = 0.001] were not significant.

Results also showed that, misperception of anger in ingroup faces was not due to a tendency to perceive more emotion in general in ingroup members. The main effects of personality type and category label, and the interaction between these two variables, all were not significant on perceptions of happiness, sadness, and fear, *F*s < 1.65, *p*s > 0.05 (see **Figure 5**).

The influences of threat management system are assumed to be manifested in many different forms of cognitive and emotional responses. Consistent with this theory, researchers found that the phenomenon of ingroup favoritism was so robust that it could be demonstrated by many different tasks, such as evaluation task, resource allocation task, attribution task, recognition task, and many other ways (e.g., Bernstein et al., 2007; Brewer, 2007; Richeson and Trawalter, 2008; Montalan et al., 2012). More importantly, the bias of ingroup favoritism was also found in the emotion perception task. Consistent with the hypothesis that the outgroup members pose greater threat of aggression than the ingroups for Westerners, researchers did find that, activating a self-protection goal led Western participants to detect greater anger in outgroup faces (than in ingroup faces) which were actually in the absence of any clear angry facial expressions (Maner et al., 2005).

Similar results were found in Study 4. In summary, the results of Study 4 indicated that, when viewing neutral faces of ingroup and outgroup members, mainland Chinese misperceived greater anger in the faces of ingroup members than in the faces of outgroup members. These results were consistent with our prediction, they demonstrated that mainland Chinese perceived greater threat of aggression from ingroup members. Therefore, results of Study 4 provide direct support to the part of our hypothesis that the greater threat of aggression was incurred by ingroup members for Chinese participants.

## Study 5

Study 4 indicates that the greater threat of aggression is perceived in ingroup faces for Chinese. But, how does this bias contribute to the ingroup derogation responses? According to our hypothesis, as a functional flexible mechanism, the response of ingroup derogation mechanism should be adjusted according to the specific perceived vulnerabilities to intragroup aggression and intergroup aggression. Therefore, the higher of the perceived aggressiveness in ingroups relative to the perceived aggressiveness in outgroups, the stronger the responses of ingroup derogation mechanism should be. In addition, as a special adaptation to a particular ecological condition in which the greater threat of aggression is brought by ingroup members, the ingroup derogation mechanism should respond more strongly to the threat of interpersonal aggression posed by ingroup members. Such ingroup derogation response should even be more exaggerated when ingroup and outgroup members are both displaying cues of aggression. We tested these possibilities in Study 5.

Since the three prior behavioral experiments had all consistently shown that the assignment of personality type was irrelevant to the results, we did not include this variable into our analysis in Study 5.

### Method

#### Participants and Design

Eighty-two paid volunteers, all mainland Chinese undergraduate students (age 18–23; 41 males and 41 females), participated in this study. A 2 (category label: ingroup, outgroup) × 2 (threat condition: aggression salient, control) within-subjects design was used.

This study was carried out in accordance with the recommendations of the IRB of the Institute of Psychology, Hunan Normal University, with written informed consent from all participants. All participants gave written informed consent in accordance with the Declaration of Helsinki.

#### Materials and Procedure

The bogus personality test and the facial stimuli employed by Study 5 were identical to those of Study 3B. Following prior research (Wu et al., 2015), half of the facial stimuli of ingroup and outgroup members were randomly labeled with a pentacle to indicate that these people had a propensity for aggression.

Participants were instructed to take the computerized personality test at first. Then they were instructed to finish the face appraisal task as described in Study 3B. Before this task, they were informed that people labeled with a pentacle had a propensity for aggression.

After the face appraisal task, participants were then asked to finish an emotion appraisal task. The procedure of this emotion appraisal task was identical to that of Study 4, except that the faces not labeled with the pentacle in the face appraisal task (i.e., 20 ingroup faces and 20 outgroup faces) were chosen as the stimuli for the emotion appraisal task.

### Results and Discussion

#### Effects of Aggression Salient Cues

**FIGURE 6:**
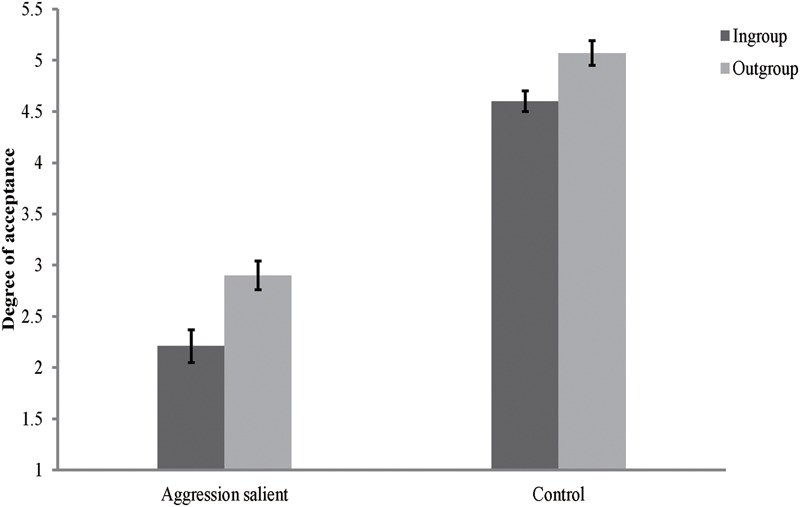
**Degree of acceptance of faces labeled as ingroup members and outgroup members in Study 5.** Error bars represent standard errors.

The rating scores of the face appraisal task were subjected to a 2 (category label: ingroup, outgroup) × 2 (threat condition: aggression salient, control) repeated-measures ANOVA. The results showed that, the main effect of category label [*F*(1,81) = 80.24, *p* < 0.001, $\eta_{p}^{2}$ = 0.5], the main effect of threat condition [*F*(1,81) = 210.43, *p* < 0.001, $\eta_{p}^{2}$ = 0.72], and the interaction between these two variables [*F*(1,81) = 4.05, *p* < 0.05, $\eta_{p}^{2}$ = 0.05] were all significant. Further simple effects analysis showed that, the aggression salient cues significantly lowered the rating scores for both ingroup members [*F*(1,81) = 232.94, *p* < 0.001, $\eta_{p}^{2}$ = 0.74] and outgroup members [*F*(1,81) = 152.68, *p* < 0.001, $\eta_{p}^{2}$ = 0.65]. The results also showed that the participants consistently derogated their ingroup members under all threat conditions [aggression salient: *F*(1,81) = 58.36, *p* < 0.001, $\eta_{p}^{2}$ = 0.42; control: *F*(1,81) = 36.08, *p* < 0.001, $\eta_{p}^{2}$ = 0.31] (see **Figure 6**).

To further illustrate the interaction between category label and threat condition, two kinds of additional composite scores were created. First, the rating scores for the faces in the control condition were subtracted by that scores of faces in the aggression salient condition to create separately a score of aggression for both ingroup and outgroup members. Then the rating scores of outgroup members in the face appraisal task were subtracted by that scores of ingroup members to separately create a score of ingroup derogation for each threat condition. Pairwise *t*-tests showed that, as predicted, the effect of aggression cues was stronger for ingroup members (*M* = 2.39, *SD* = 1.42) than for outgroup members (*M* = 2.17, *SD* = 1.59), *t*(81) = 2.01, *p* < 0.05, and the participants exaggerated their ingroup derogation attitudes even when both ingroup and outgroup members were displaying the propensity for aggression (aggression salient: *M* = 0.7, *SD* = 0.54; control: *M* = 0.51, *SD* = 0.62), *t*(81) = 4.25, *p* < 0.001.

#### Perceived Relative Aggressiveness and Ingroup Derogation

**FIGURE 7:**
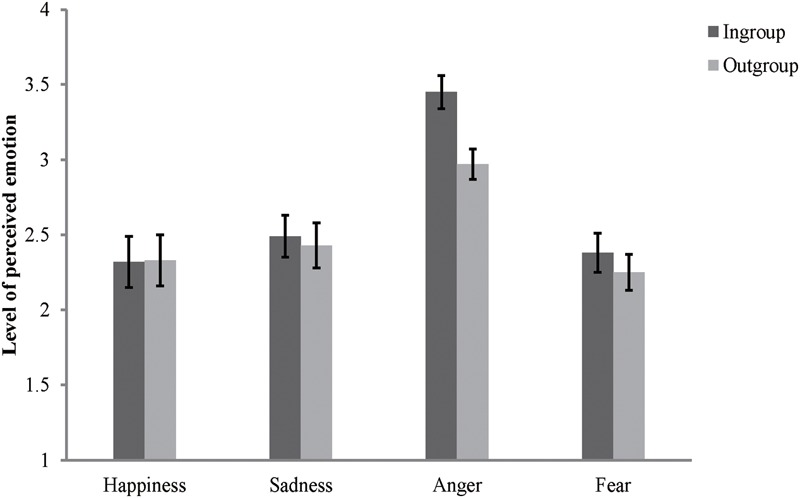
**Mean levels of perceived emotions in inroup and outgroup faces in Study 5.** Error bars represent standard errors.

Consistent with Study 4, participants perceived greater threat of aggression from ingroup members. Pairwise *t*-test showed that more anger was perceived in ingroup faces than in outgroup faces, *t*(81) = 6.73, *p* < 0.001, but no such effect was observed in the perception of happiness, *t*(81) = -0.33, *p* > 0.05, and sadness, *t*(81) = 1.08, *p* > 0.05 (see **Figure 7**). The results also showed that participants perceived more fear in ingroup faces than in outgroup faces, *t*(81) = 2.94, *p* < 0.01. This unexpected result on fear perception can be explained by a functional projection process (Maner et al., 2005). As a direct response to the threat of aggression, fear facilitates the organism to escape from the impending dangers and recognizing the facial expressions of fear in others can also help an individual to detect the potential threats (Shariff and Tracy, 2011). Given that the face appraisal task had directly primed the participants with cues of aggression (especially from ingroups) and the social interactions usually occur within the boundary of ingroups (Schaller and Neuberg, 2012), identifying these non-aggressive ingroup members with a facial expressions of fear would help an individual to detect the potential threats of aggression posed by these aggressive ingroup members. Therefore, this result is still compatible with our hypothesis.

To examine the relationship between perceived relative aggressiveness and ingroup derogation, the rating scores of facial expressions on ingroup faces were separately subtracted by those scores of outgroup faces to create four composite emotion scores (happiness/sadness/anger/fear). As predicted the results of Pearson product-moment correlations revealed that, only the perceived relative aggressiveness (the composite emotion score of anger) was positively correlated with ingroup derogation attitudes (ingroup derogation scores). There were no significant associations between the other three composite emotion scores and the ingroup derogation attitudes (as shown in **Table 1**).

**Table 1:** The correlations between the composite emotion scores and ingroup derogation.

Ingroup derogation	Happiness	Sadness	Anger	Fear
Aggression salient	0.12	0.18	0.55^∗∗∗^	0.09
Control	0.15	0.19	0.57^∗∗∗^	0.01

*^∗∗∗^*p* < 0.001.*

In summary, Study 5 yielded results consistent with our predictions. The results showed that, the ingroup derogation mechanism separately responded to the aggression cues mediated by ingroup and outgroup members, and a stronger response was given to the aggression cues mediated by ingroup members. An exaggerated ingroup derogation attitude was also found when both the ingroup and outgroup members were displaying the propensity for aggression. In addition, the results also revealed that the perceived relative aggressiveness and ingroup derogation attitudes was interconnected. Taken together, these results suggest that ingroup derogation is a functional flexible mechanism that can adjust its response according to the specific perceived vulnerabilities to intragroup aggression and intergroup aggression.

## General Discussion

Across five different studies, we consistently found the evidence that support our hypothesis. The results of computer simulations (Study 1) showed the possibility for the evolution of an ingroup derogation tendency due to escalated intragroup aggression. The results obtained by behavioral experiments (Studies 2 and 3) further showed that, as it should be for an evolved threat management mechanism, the ingroup derogation mechanism followed the smoke detector principle (i.e., responding to heuristic cues which implied the differentiation between “us” and “them”) and the functional flexibility principle (i.e., responded more strongly when participants subjectively felt vulnerable to interpersonal aggression or when there were contextual cues of interpersonal aggression in the immediate environment). The results also revealed that the ingroup derogation mechanism separately responded to the aggression cues mediated by ingroup and outgroup members, and a stronger avoidance response was elicited by the aggression threat incurred by ingroup members. An exaggerated ingroup derogation attitude was also found when both the ingroup and outgroup members were displaying the tendencies for aggression (Study 5). These results are consistent with our hypothesis that the activation of ingroup derogation is related to an ecological condition in which the greater threat of aggression is incurred by ingroup members. Further evidence consistently revealed that Chinese participants did perceive their ingroup members as more aggressive (Study 4) and such a bias was positively interconnected with the ingroup derogation attitudes (Study 5). Collectively, the current results suggest that ingroup derogation is related to an evolved psychological mechanism designed to deal with a special ecological condition in which the greater threat of aggression is incurred by ingroup members.

As an evolved threat management system, the ingroup derogation mechanism should be prone to make false-positive errors (i.e., smoke detector principle). However, such a threat management mechanism shouldn’t be so responsive that it responds to any other unspecific cues to the potential threat since that would be too costly and even may interfere with other fitness-enhancing activities (Van Vugt and Park, 2009; Neuberg et al., 2011; Schaller and Neuberg, 2012; Cottrell and Park, 2013). Consistent with this theory, in Study 5, we found that only the perceived relative aggressiveness was positively correlated with ingroup derogation attitudes. It should be noted at here, although the functional projection phenomenon was also observed in the perception of fear, as a direct response to the presence of potential danger but a non-indicative cue to one’s aggressiveness (i.e., the facial expression of fear; Shariff and Tracy, 2011), the composite score of fear was not significantly correlated with ingroup derogation attitudes in Study 5. These results suggest that the perceived greater aggressiveness in ingroups (relative to outgroups) should be one of the most relevant cues for triggering ingroup derogation attitudes. Similar results were also found in the previous studies of ingroup favoritism. These studies have suggested that the perceived greater aggressiveness in outgroup members (relative to ingroup members) is directly related to the ingroup favoritism tendencies (Van Vugt and Park, 2009; Schaller and Neuberg, 2012; Cottrell and Park, 2013). For example, researchers found that the more likely a white female judges a black male to be physically near (thus more likely to be harmed), the more likely she evaluates this black male as negative (Cesario and Navarrete, 2014). Taken together, these results suggest that the perceived aggressiveness between groups should be one of the key moderators between ingroup favoritism and ingroup derogation. It should be noted that, in the current study, the pattern found between perceived aggression and ingroup derogation was fully correlational. To completely illustrate the casual links between perceived aggressiveness and ingroup derogation attitudes, researchers still need to differentially manipulate the likelihood of aggression created by ingroup and outgroup members. Nevertheless, one recent study did find that, if the intragroup aggression was made to be the major threat, participants would be more willing to harm their ingroups instead of outgroups (Barker and Barclay, 2016). Since the manipulation of aggression was confounded with the scale of competition within that study, their results are still not conclusive. More direct tests are needed. This would be a very important direction for future studies.

Is ingroup derogation driven by ingroup avoidance or it is driven by outgroup attraction? While previous study showed that ingroup derogation could be driven by ingroup avoidance and outgroup attraction at the same time (but it depends on the source of the disease threat; see Wu et al., 2015), we provide limited answers to this question in the current study. The contextual cues of interpersonal aggression had no significant effects either on the ingroup attitudes or outgroup attitudes in Study 3, but the results of Study 5 suggest that it is mainly driven by ingroup avoidance (i.e., the avoidance tendencies were more exaggerated when the ingroup members were displaying cues of aggression). Since the attractiveness or the acceptance for faces is affected by many different factors (e.g., Rhodes, 2006; Thornhill and Gangestad, 2006; Little et al., 2011), it is not surprising that the Study 3 failed to differentiate the effect of contextual cues of aggression from the individual differences by employing the between-subjects design. Will the threat of aggression has the same effect on the ingroup derogation attitudes as the threat of disease? Theories have been proposed that a threat management system should be functional specific in that it should respond differentially to different kinds of threats (Van Vugt and Park, 2009; Neuberg et al., 2011; Schaller and Neuberg, 2012; Cottrell and Park, 2013). Therefore, it might be expected that the effect of aggression is different from the effect of disease on ingroup derogation. More rigorous tests employing more reliable dependent measures (such as IAT or the startle reflex; e.g., March and Graham, 2015) are needed before clear conclusions can be drawn.

Although we proposed that our hypothesis could also explain the ingroup derogation found among minority groups, the current results provide no evidence to support this claim. However, previous studies suggest that this claim could be supported. For example, March and Graham (2015) found that Hispanic women and White women both displayed startle eye blink reflex and IAT responses indicative of negative attitudes toward Hispanic male faces relative to White male faces. Since the startle eye blink reflex response could be seen as a kind of fear response (Phelps et al., 2000), and fear is assumed to be an adaptive emotion designed by natural selection to facilitate escape from or to defense against the threat of aggression (Schaller and Neuberg, 2012), the study of March and Graham (2015) suggests that the Hispanics perceive more threat of aggression from their ingroup members while they are endorsing the negative attitudes toward their ingroup members. More direct empirical evidence is needed considering that this evidence is indirect and preliminary.

It should also be noted that we derived our hypothesis primarily from an evolutionary perspective. The present studies were not designed to directly examine the exact psychological structures or the processes of ingroup derogation, nor were they designed to offer any explanations in terms of proximate cause. They only intended to offer a functional explanation for the ingroup derogation phenomenon. Since the ultimate and proximate explanations are not exclusive to each other and both are essential to our understanding of human behaviors (Laland et al., 2011; Scott-Phillips et al., 2011), researchers still need to directly examine the proximate mechanisms of ingroup derogation in the future.

In summary, the current results provide the evidence that can support our hypothesis. They suggest that except of being an adaptive response of the behavioral immune system (Wu et al., 2015), ingroup derogation may also be related to another threat management system that is designed to deal with a special ecological condition in which the greater threat of aggression is incurred by ingroup members. The current study indicates a potential causal link between the threat of intragroup aggression and the ingroup derogation attitudes.

## Author Contributions

QW and PZ conceived and designed the experiments. QW, WL, CL, and XL performed the experiments. QW, WL, and CL analyzed the data. QW drafted the paper. All the authors participated in the revising of the paper. All the authors have approved of the version’s publishment and agreed to be accountable for all aspects of the work.

## Conflict of Interest Statement

The authors declare that the research was conducted in the absence of any commercial or financial relationships that could be construed as a potential conflict of interest.

## Footnotes

1 https://www.cia.gov/library/publications/the-world-factbook/index.html

2 http://www.gallup.com/poll/150464/americans-believe-crime-worsening.aspx

3 In Study 3B, we used the degree of acceptance (i.e., acceptance of a specific group member as a partner to work with) as the measure of participants’ preference for a specific group member (for a similar measure of ingroup favoritism, see Navarrete and Fessler, 2006). Previous studies of ingroup favoritism have shown that participants incline to affiliate to and cooperate with their ingroup members rather than with outgroup members (e.g., Navarrete and Fessler, 2006; Ruﬄe and Sosis, 2006; Rand et al., 2009; Yamagishi and Mifune, 2009). Studies on ingroup derogation also showed that the Chinese are more inclined to choose an outgroup member as a partner to work with (Wu et al., 2015).
