# Supplementary material for: A Price Paid for Our Internal Strife: Escalated Intragroup Aggression and the Evolution of Ingroup Derogation
Source: Front Psychol. 2016 Sep 22;7:1453. doi: 10.3389/fpsyg.2016.01453 (PMC5031712; doi:10.3389/fpsyg.2016.01453)
Supplement: Supplementary file 1 [file Presentation_1.PDF]

Supplementary Material

A Price Paid for Our Internal Strife: Escalated Intragroup Aggression and the Evolution of Ingroup Derogation

Qi Wu, Wang Liu, Chen Li, Xiongfeng Li, Ping Zhou*

*** Correspondence:** Ping Zhou: pingzhouhunnu@gmail.com

1 The Model

The asexual Penna model implements the mutation accumulation process by dividing life into A_{max} time intervals and by representing the chromosomes through a string of A_{max} bits, each of which can be zero or one. A zero bit means health, a bit set to one means an inherited disease starting to act from that age on which corresponds to the position of the bit-string. If T bits are active, their combination kills the individual. Each individual which has reached the minimum reproduction age R gets B offspring in each year. The offspring inherits the mother's bit-string except for M mutations introduced during this process. The sexual Penna model inherits all the characteristics of the asexual Penna model, except that the chromosome of each individual is represented by a pair of bit-strings, one coming from the mother and the other from the father. These two bit-strings are read in parallel. The offspring chromosome is constructed by randomly crossing the bit-strings of one of its parents, and choosing one of the two remaining strings to be the offspring one. Mutations are also introduced during this process. The same procedure is repeated for the bit-strings of the other parent, generating the second string of the offspring. See Penna (1995) and Sá Martins and Stauffer (2001) for the original description of the asexual and sexual Penna models.

In Study 1, the classic sexual Penna model was slightly modified and placed on a lattice to simulate the evolution of ingroup derogation.

1.1 Representation of the Virtual Individual

The virtual population is divided into males and females, with N denoting different social groups in total. Each virtual individual is represented by three "chromosomes", which consists of six bit-strings of size A_{max}. Therefore, each chromosome is represented by two bit-strings. Specifically, we have the Health Bit-String to control the virtual individuals' health and the Ingroup/Outgroup Bit-String to control the virtual individuals' attitudes toward ingroup or outgroup members (as shown in Supplementary Figure 1).

1.1.1 Health Bit-String

In each time step, one new position of all virtual individuals' Health Bit-String will be read in parallel. If a virtual individual has two bits equal to 1 in the same position of Health Bit-String at that time step, it will start to suffer the effects of an inherited disease from that time step ("year") until its death. If at any age a virtual individual has already acquired T diseases, it dies at that year. Since the size of

strings is $A_{\max}$, each virtual individual can live at most for $A_{\max}$ years (i.e., it dies when it reaches the age $A_{\max}$).

1.1.2 Ingroup/Outgroup Bit-String

These bit-strings independently (and only) control a virtual individual's ingroup and outgroup attitudes from its birth. All contents of these bit-strings are read in parallel. Therefore, an individual displays a constant attitude toward ingroup or outgroup members in its lifetime. If a virtual individual has two bits equal to 1 at the *i*-th position of these bit-strings, its avoidance tendency toward ingroup or outgroup members is "strong" at position *i*. The number of homozygous positions with two bits "1" of the Ingroup and Outgroup Bit-String weighs the avoidance tendency toward ingroup and outgroup members (respectively, F_{in} for ingroup members, and F_{out} for outgroup members).

If $F_{in} > F_{out}$, it means the virtual individual prefers outgroup members over its ingroup members.

If $F_{out} > F_{in}$, then it means the virtual individual prefers ingroup members to outgroup members. The avoidance tendency toward ingroup members is given by:

$$F_{in} = \frac{\sum_{i=1}^{A_{\max}} B(i)}{A_{\max}} \quad (1)$$

where $B(i)$ indicates the presence of strong avoidance tendency for Ingroup Bit-String with position i.

The avoidance tendency toward outgroup members for an individual is also determined in this way:

$$F_{out} = \frac{\sum_{i=1}^{A_{\max}} \mathrm{C}(i)}{A_{\max}} \quad (2)$$

where $\mathrm{C}(i)$ indicates the presence of strong avoidance tendency for Outgroup Bit-String with position i.

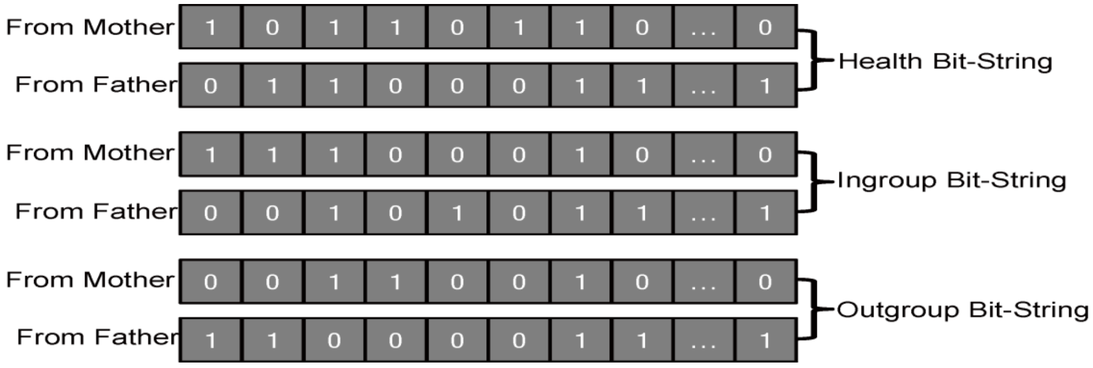

Supplementary Figure 1. An individual is represented by six bit-strings. The Health Bit-String determines the individual’s health condition, while the Ingroup and Outgroup Bit-String determines its avoidance tendency toward ingroup and outgroup members.

1.2 Virtual Continent

Each virtual individual lives on a given site (i, j) of a $L \times L$ square lattice with periodic boundary conditions (i.e., the right side is connected to the left side of the lattice, and the top is connected to the bottom of lattice, just like a round tube with its head connected to its tail). Each site can only contain maximum amount of $\mathrm{Re}_{\max}$ resources vital for survival. In each year, a virtual individual consumes the resources at rate of Re_{con} and dies if it cannot gain access to that resource (i.e., the amount of resources within its current site is less than Re_{con}). For an empty site, the amount of resources increases at a rate of Re_{inc} in each year.

1.3 Intragroup and Intergroup Aggression

Because resources on the virtual continent are limited, individuals who cannot gain access to the necessary resource Re_{con} will try to take another site in order to survive. It randomly moves into one of the suitable sites (resource $\geq \mathrm{Re}_{con}$) among its eight neighboring sites. If no such sites are available, it stays at its own site. If the neighboring site is not occupied by any individual, the invader will simply take this empty site. But, if the neighboring site is occupied, conflicts will occur. The original owners will defend themselves and fight back. The invader can avoid such conflicts according to its intragroup and intergroup attitudes, otherwise it has to engage in a battle with the defender. Invader avoids ingroup defenders with probability of F_{in} and avoids outgroup defenders with probability of F_{out}. The Invaders have a probability of P_{in} or P_{out} to lose the battle (thus $1-P_{in}$ or $1-P_{out}$ to win the battle) according to defenders' social group (P_{in} for ingroup members, P_{out} for outgroup members). Higher value in P_{in} indicates greater level of intragroup aggression, whereas higher value in P_{out} indicates greater level of intergroup aggression. Losers of such conflicts die instantly. If invader successfully avoids a conflict, it returns to its own site. Then the individual randomly moves into another unexplored suitable sites around its own site again (and the subsequent conflicts and avoidance happens thereafter). Such a process continues until the individual successfully takes a new site to live or until the individual dies during the fight. But, if all suitable sites are explored and the individual is still staying at its own site, it will stay at that site and will not try to move again.

1.4 Reproduction

If a female succeeds in surviving until the minimum reproduction age R, she randomly chooses a male whose age must also be no less than R among her eight neighboring sites to mate. A female who fails to find such an available male cannot reproduce. If the female succeeds, she generates one offspring and randomly chooses an empty site from her eight neighboring sites to place the baby. The newborn dies if there is no site available.

The offspring's chromosomes are constructed from the parents'. First the mother's strings are randomly crossed and three female gametes are produced. M random mutations are then introduced (i.e., M positions within the gametes are randomly chosen, then the logical NOT operator is applied to their contents). The same process occurs with the father's genomes, and the union of these six genomes forms the genomes for an offspring (see Supplementary Figure 2). The sex of the newborn is randomly chosen. The offspring randomly joins one of the social groups of its parents.

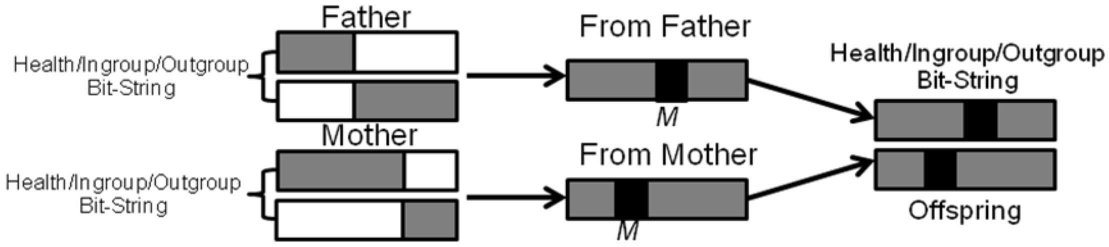

Supplementary Figure 2. The construction of offspring's genomes in the process of sexual reproduction.

1.5 Order of Events

At every time step, the events occur in a given order: first, the virtual individual grows and ages; then the virtual individual consumes the resources and the resources increase on the empty sites; third, the virtual individual try to take others' sites if the condition of short of resources is satisfied; finally, the virtual individual reproduces. All these processes are applied over the whole virtual population and over all time steps.

2 Simulation Protocol

Simulations were initialized by randomly distributing N_0 virtual individuals on a square lattice. Both the sex and the social group of a virtual individual were randomly initialized. The Health Bit-String was initialized with all the positions equal to zero. The contents of Ingroup and Outgroup Bit-String were also randomly generated. To make the model tractable, following parameters were fixed at the reasonable values: $L = 100, \mathrm{N}_0 = 7500, A_{\max} = 100, \mathrm{Re}_{\max} = 40, \mathrm{Re}_{con} = 2, \mathrm{Re}_{inc} = 8, R = 11, T = 7, M = 1$. Since for our ancestors the mean number of group members in a group was about 150 (Dunbar, 1993), the number of social groups N was initialized to be 50 (i.e., $N_0 / N = 7500/50 = 150$).

Simulations were carried out 20 times for a given set of parameters using different initial seeds for the random number generator, and were stopped when the virtual system had converged (i.e., the order parameters had stabilized around certain stationary values in each round of simulations, including the population size and the mean avoidance tendencies toward ingroup or outgroup members).

3 Results of Population Size

The mean population size of the 20 simulations was taken as the results of population size for a given set of parameters. For all conditions, the artificial species survived on the lattice, with a very stable population size around 7300. Detailed results are shown in the following figures.

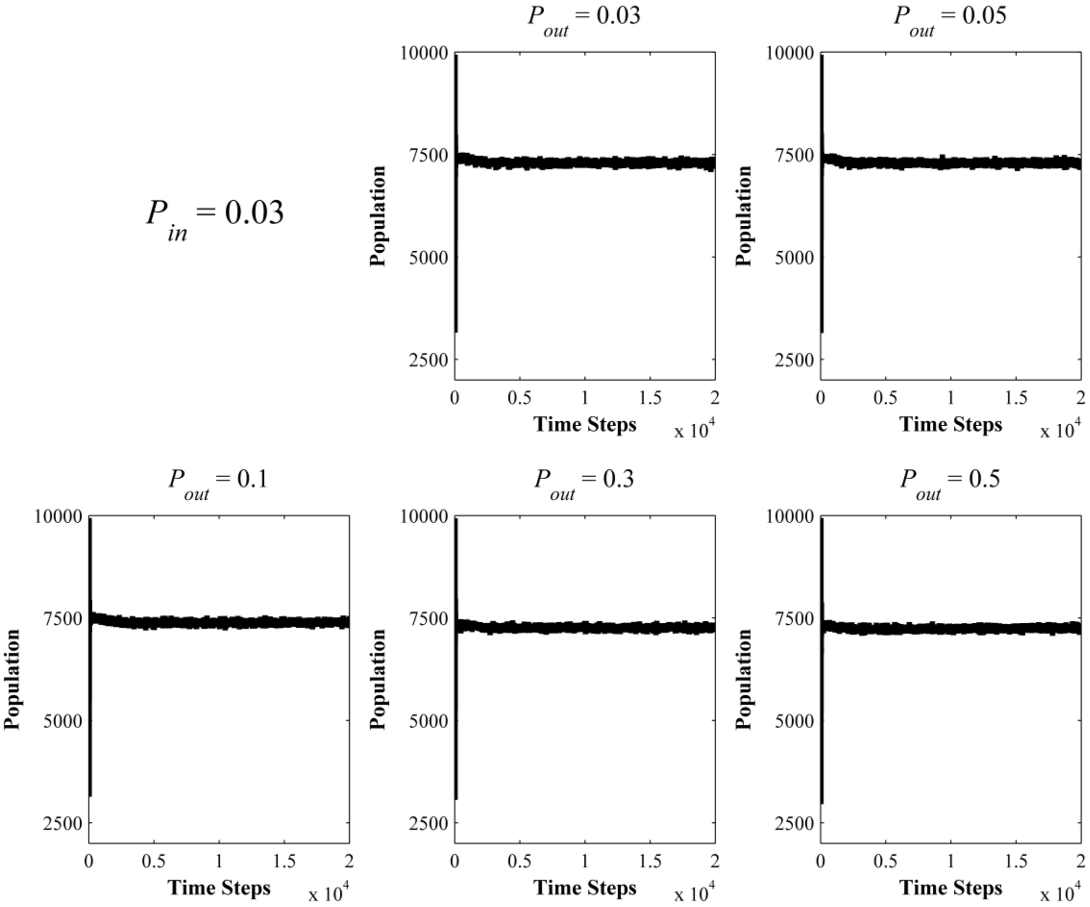

Supplementary Figure 3. Population size as a function of time with $P_{in} = 0.03$. The ordinate axis denotes population size, while the horizontal axis represents the time steps.

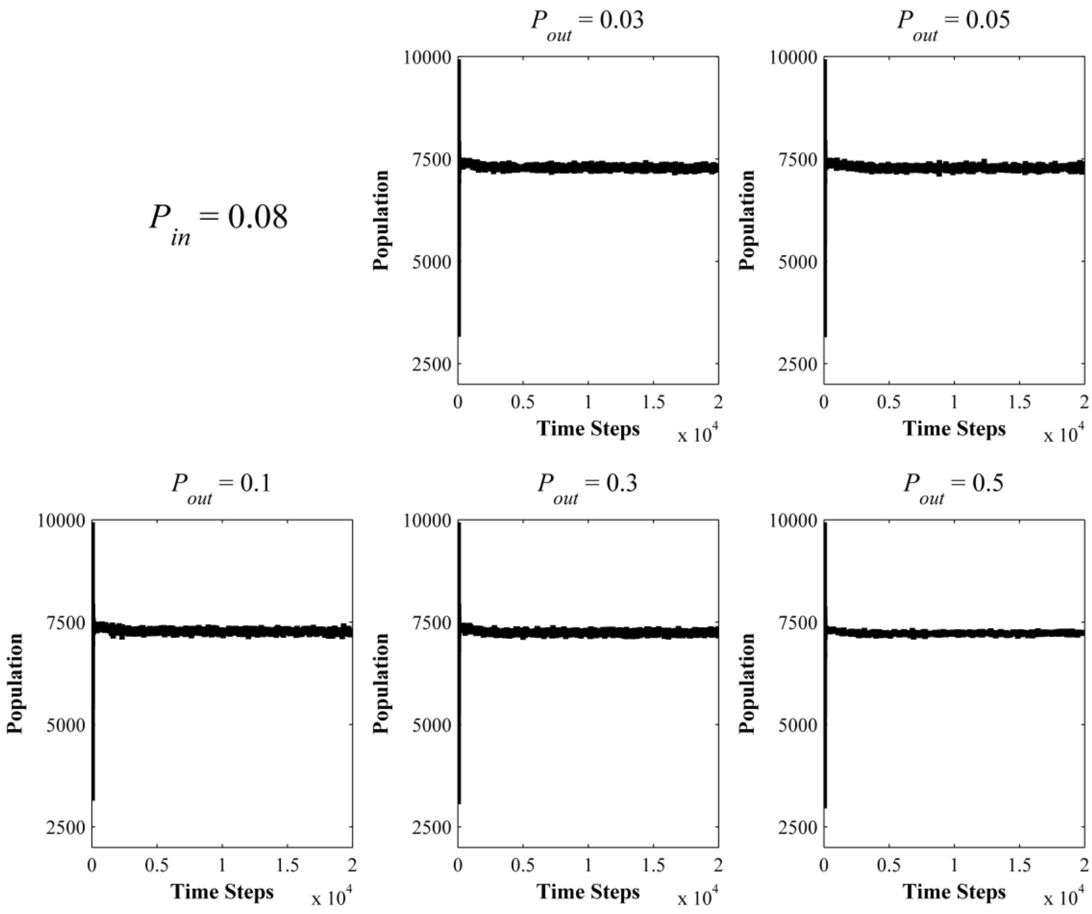

Supplementary Figure 4. Population size as a function of time with P_{in} = 0.08 . The ordinate axis denotes population size, while the horizontal axis represents the time steps.

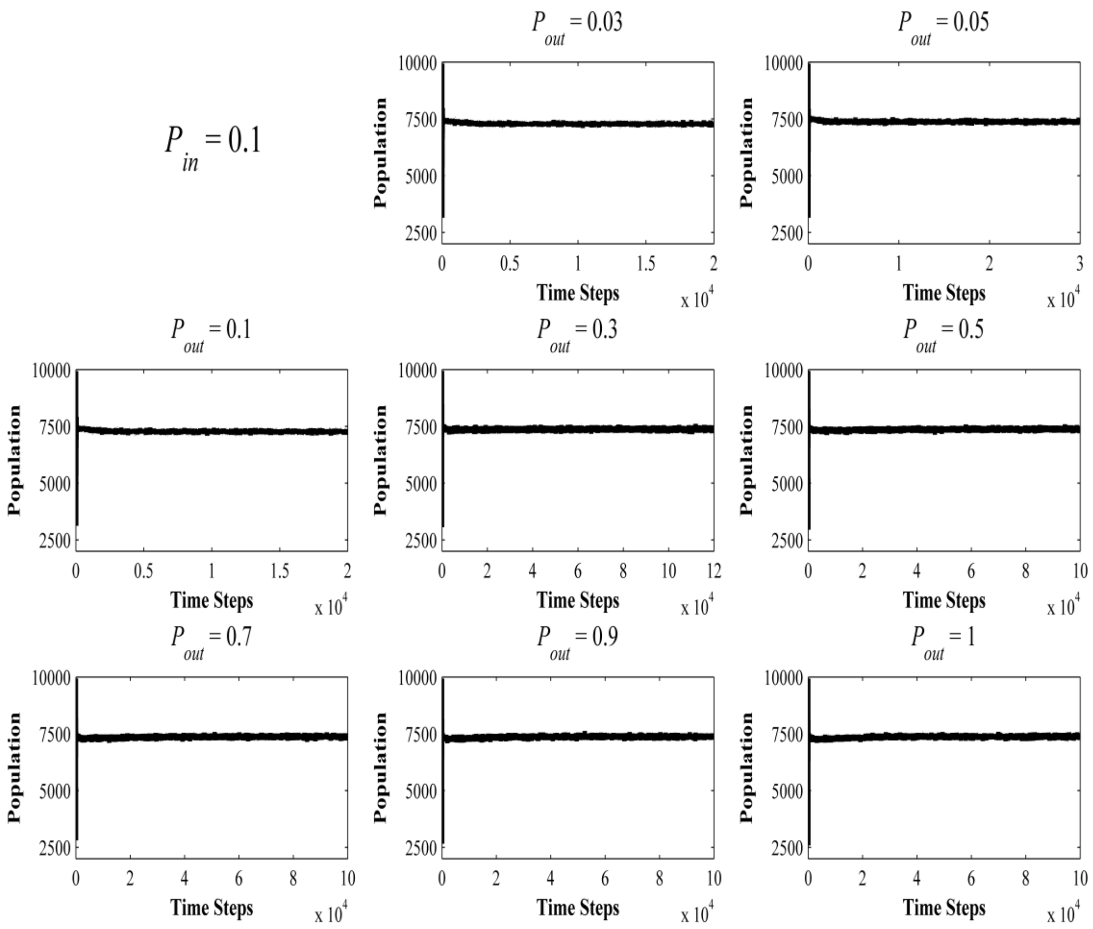

Supplementary Figure 5. Population size as a function of time with $P_{in} = 0.1$. The ordinate axis denotes population size, while the horizontal axis represents the time steps.

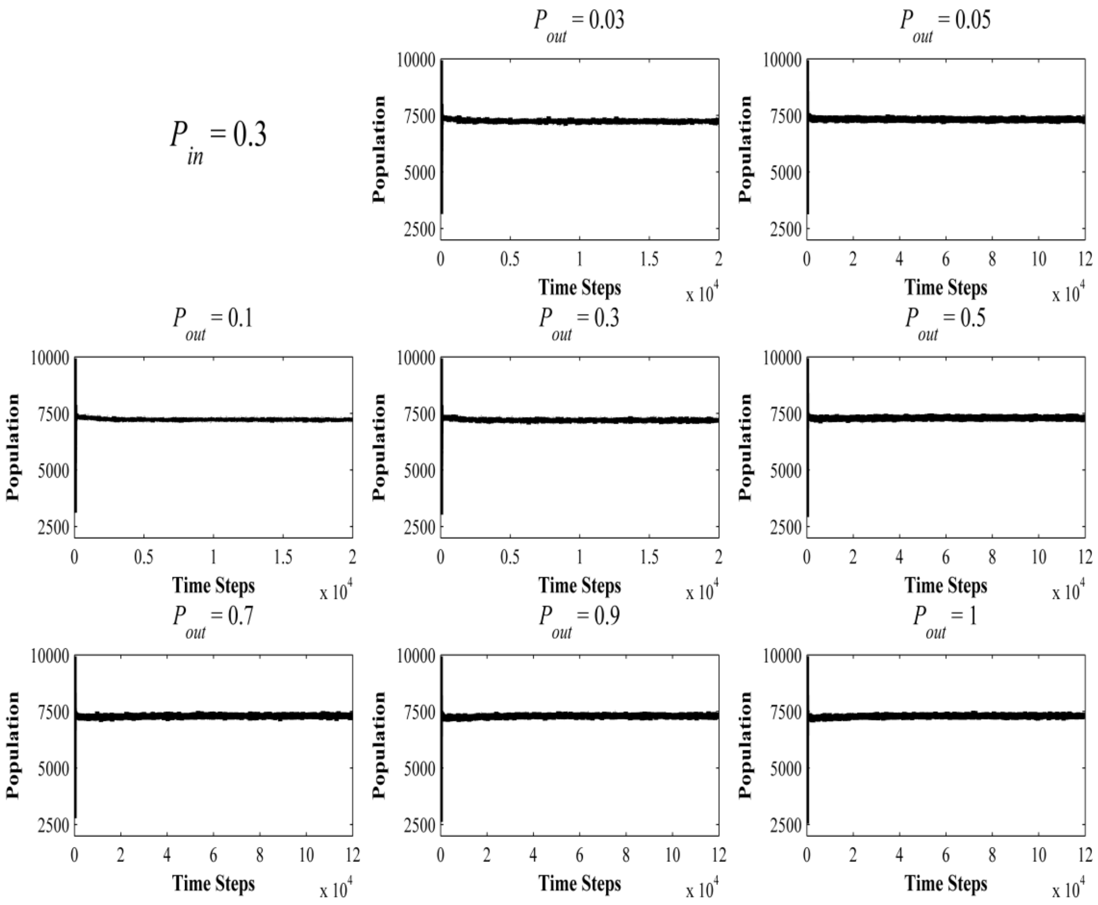

Supplementary Figure 6. Population size as a function of time with $P_{in} = 0.3$. The ordinate axis denotes population size, while the horizontal axis represents the time steps.

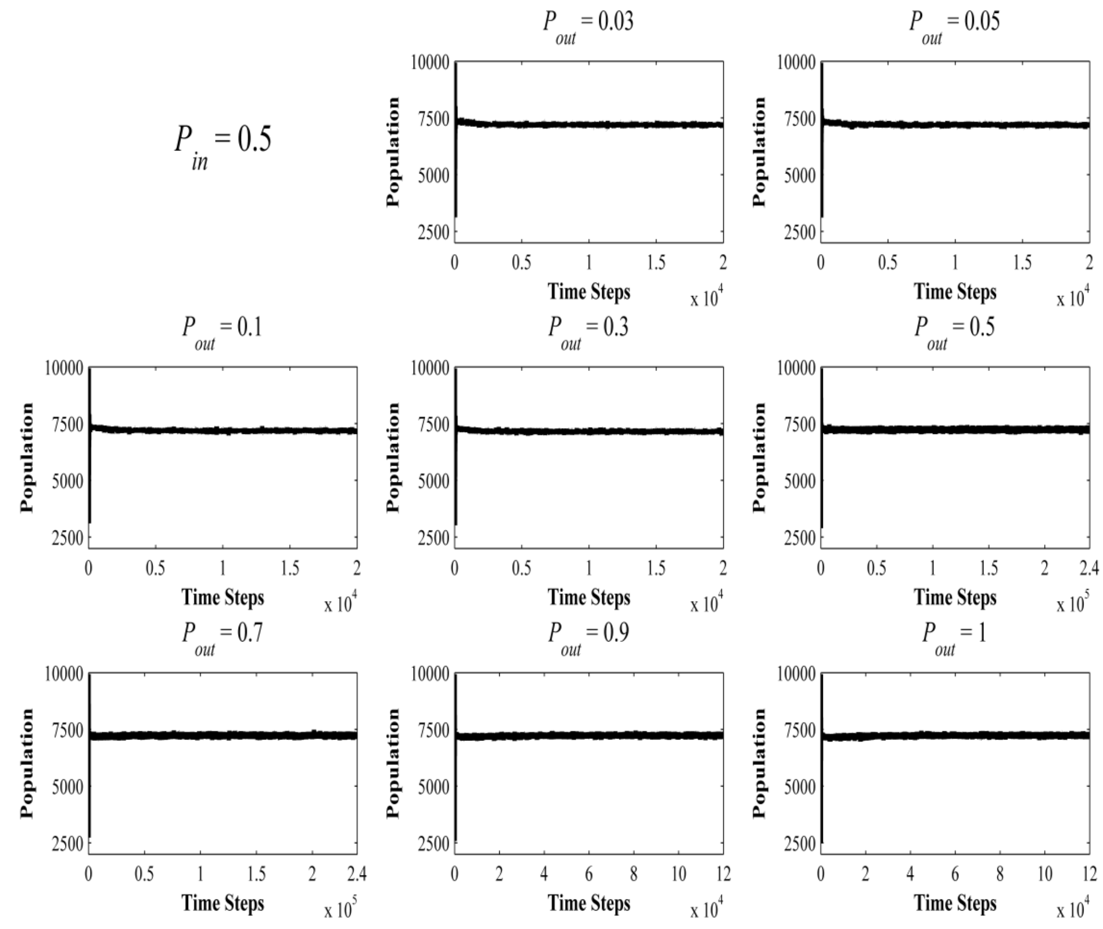

Supplementary Figure 7. Population size as a function of time with $P_{in} = 0.5$. The ordinate axis denotes population size, while the horizontal axis represents the time steps.

References

Dunbar, R. I. M. (1993). Coevolution of neocortical size, group size and language in humans. *Behav. Brain. Sci.* 16, 681–735. doi:10.1017/S0140525X00032325

Penna, T. J. P. (1995). A bit-string model for biological aging. *J. Stat. Phys.* 78, 681–735. doi: 10.1007/BF02180147

Sá Martins, J. S., and Stauffer, D. (2001). Justification of sexual reproduction by modified Penna model of ageing. *Physica. A*. 294, 191–194. doi:10.1016/S0378-4371(01)00127-3
